# Comparison Between Deltascan Single Channel Electroencephalography (EEG), Confusion Assessment Method-Intensive Care Unit (CAM-ICU) Score and Clinical Assessment in Diagnosing Delirium in Intubated Patients in the Intensive Care Unit

**DOI:** 10.7759/cureus.26449

**Published:** 2022-06-30

**Authors:** Juul Aben, Sjaak Pouwels, Annemarie Oldenbeuving

**Affiliations:** 1 Critical Care, Elisabeth-Tweesteden Hospital, Tilburg, NLD; 2 Intensive Care Medicine, Elisabeth-Tweesteden Hospital, Tilburg, NLD; 3 Intensive Care Unit, Elisabeth-Tweesteden Hospital, Tilburg, NLD

**Keywords:** complications, cam-icu, intensive care, monitoring, electroencephalography, delirium

## Abstract

Background

The aim of this article is to assess the feasibility of using single-channel electroencephalography (EEG) measurement for detecting delirium in intubated Intensive care (ICU) patients and to assess the level of agreement between the EEG measurements, the CAM-ICU score and the clinical diagnosis of delirium.

Materials and methods

This study was an exploratory pilot between May 2021 and September 2021 including intubated patients in the ICU. For this study the Prolira^®^ (Arnhem, The Netherlands) Deltascan single-channel EEG was used and compared with the Confusion Assessment Method (CAM)-ICU and the clinical diagnosis of delirium by ICU physicians.

Results

In total 23 patients were found eligible for this study, of which 20 were included in the final analysis. The patients mean age was 63.0 ± 8.8 years, and the majority (thirteen) was male (65%). In total 17 of the 20 patients (85%) received the diagnosis delirium by the medical team. There were no statistically significant differences between the Deltascan and CAM-ICU measurements in diagnosing delirium per time point (p values respectively 0.21; 0.90; 0.34; 0.11; 0.056 and 0.091). AUCs for the agreement between the CAM-ICU and the Deltascan measurements were respectively: 0.676 ± 0.205; 0.333 ± 0.224; 0.402 ± 0.146; 0.488 ± 0.202; 0.06 ± 0.077 and 0.06 ± 0.109 (all p>0.05). AUCs for the level of agreement between the clinical diagnosis delirium and Deltascan were: 0.676 ± 0.152; 0.686 ± 0.146; 0.711 ± 0.132; 0.688 ± 0.136; 0.500 ± 0.158 and 0.700 ± 0.211 (all p>0.05).

Conclusion

In this exploratory study, we showed that there is no statistical agreement between CAM-ICU and Delta scan measurements. Secondly, there is a higher agreement, although not statistically significant between the clinical diagnoses of a delirium (by a clinician) with the Deltascan measurements. Despite this small study we think that the Deltascan can be of additional value in intubated ICU patients and therefore larger studies are needed to substantiate our findings.

## Introduction

In general, delirium is a common phenomenon in hospitalised patients, particularly after surgery [1-3] and in the Intensive Care Unit (ICU) [4-7]. It has been associated with prolonged hospitalisation [8-10], cognitive disturbances and long-term cognitive decline [4,9,11], increased mortality, and healthcare costs [9,12]. Especially in the ICU, its prevalence can vary between 11% to 89%, depending on the patient population [4-7]. This indicates that delirium is a serious health problem and its early detection is essential. However, intensivists and ICU nurses often underestimate the clinical occurrence of delirium [13] and therefore a more objective measurement is needed. One of these measurement tools is the Confusion Assessment Method (CAM)-ICU [14]. Several studies showed that the sensitivity of the CAM-ICU in diagnosing delirium may depend on several factors, of which patient groups and the nursing team are the most important ones [14-16]. It should be noted that irrespective of the patient population and training of nurses, the sensitivity of diagnosing delirium varies between 40% and 80% [14-16].

Delirium is associated with specific electroencephalogram (EEG) patterns, such as the slowing of background activity and polymorphic delta waves [17,18]. More recently, Numan et al. [18] showed that delirium could be adequately assessed using a single channel EEG looking at delta waves, which are associated with delirium. However, this method has not been described used in intubated patients. The aim of this exploratory pilot study will be twofold: 1) to assess the feasibility of using a single channel EEG measurement for detecting delirium in intubated ICU patients and 2) to assess the level of agreement between the EEG measurements, the CAM-ICU score and the clinical diagnosis of a delirium in intubated ICU patients.

## Materials and methods

This study was approved by the Medical Ethics Committee (METC) of the Elisabeth-Tweesteden Hospital in Tilburg, The Netherlands (METC number: NW2021-17) and adheres to the principles of the declaration of Helsinki. Either written or oral informed consent was obtained of all patients included.

Patient population and recruitment

Between May 2021 and September 2021, patients were included based on the following criteria: 1) aged 50 years and older; 2) at least 24 hours of ICU admission and 3) intubated on the first day of the study measurements. To be able to measure delta waves using a single-channel EEG, all included patients were required to have a sedation level (using the Richmond Agitation Sedation Scale [RASS]) between -3 and +4. These sedation levels were chosen because when patients are deeply sedated (RASS -4 or -5) delta waves cannot be adequately measured using the Deltascan and the CAM-ICU cannot be used [17]. Patients that were admitted to the ICU because of a cardiac arrest and/or recent neurological events (for example a cerebrovascular accident [CVA], neurotraumatology or post-anoxic encephalopathy) were excluded.

Outcome measurements

Deltascan Single-Channel EEG

**Figure 1:**
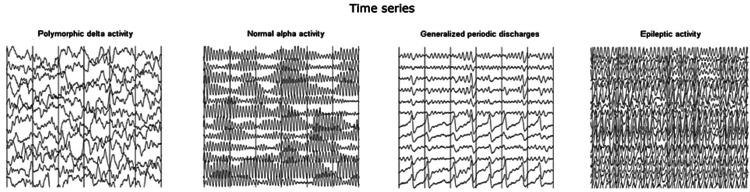
Graphical depiction of polymorph delta waves Network model of normal activity, PDA, periodic discharges, and epileptic activity.

**Figure 2:**
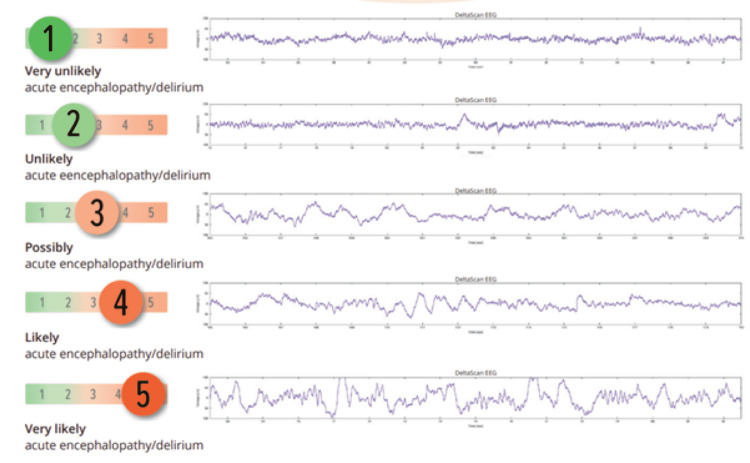
Polymorphic delta waves expressed in figure, measured by the delta scan

For this study, the Prolira® (Arnhem, The Netherlands) Deltascan single-channel EEG was used. This device was developed according to earlier EEG investigations done by van der Kooi et al. [17] with the aim to further simplify and optimise bedside EEG use. This is especially valuable because a 32-channel EEG can be very costly and time-consuming to perform. The Deltascan is a single-channel EEG device, which uses an optimal electrode combination for the detection of polymorph delta waves, which are associated with delirium or acute encephalopathy (Figures 1, 2).

**Figure 3:**
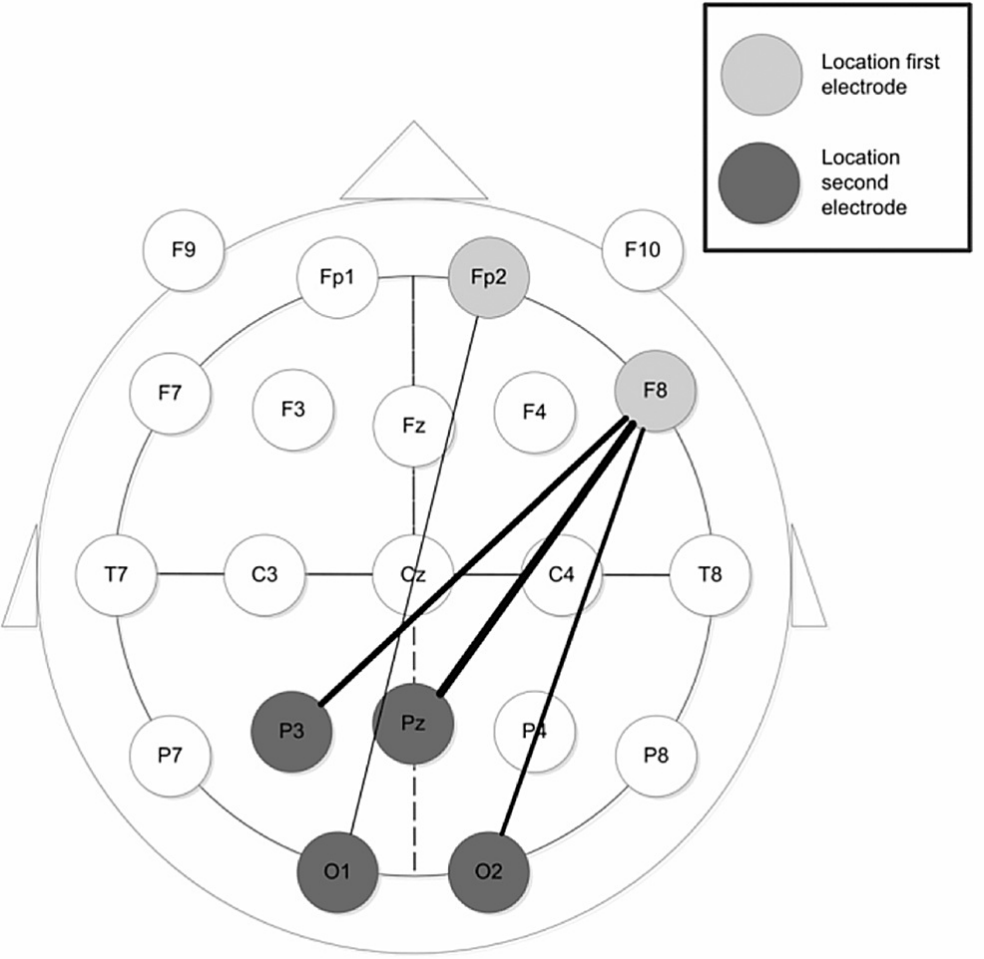
Overview of the electrode placement using the Deltascan Figure adapted from van der Kooi et al. [17].

Figure 3 gives and overview of the electrode placement using the Deltascan. Depending on the amount of polymorph delta waves measured, a score will be calculated, indicating the probability of a delirium. The score can vary from 1 (very unlikely a delirium) to 5 (very likely a delirium).

Delirium and Sedation Assessment by Nurses and Doctors

As part of their daily routine, ICU nurses in our hospital used the Confusion Assessment Method (CAM)-ICU [14,16] once every eight hours. They used the previously validated Dutch version of the CAM-ICU and reported the score in the medical records of the patients [19]. Assessments were done by bedside at the start of the shift (day-, evening- or nightshift). The level of consciousness/sedation was measured using the RASS [20].

ICU doctors (attending physicians and residents) determined the presence of a delirium based on their own clinical assessment, taking into account the general Dutch guidelines and the Diagnostic and Statistical Manual of Mental Disorders (DSM-5) criteria [21]. These assessments were also described in the patients’ medical records.

Study procedure and data collection

Author JA was trained by an expert physician affiliated with Prolira® in the use of the Deltascan equipment. The same expert physician supervised the first measurements, and was available for troubleshooting in case of device related questions and/or problems.

Author JA included each patient and the measurements with the Deltascan and CAM-ICU measurements were performed at either 08:00, 15:00 and/or 23:00 hours, with a maximum of two measurements per day. At the same time, as part of regular ICU care, the ICU nurse also took the CAM-ICU measurements and reported these in the medical records. Patient characteristics that were derived from the medical records, were diagnosis, age, gender, length, weight, body mass index (BMI), and sedative medication use.

All attending doctors and nurses were blinded for the study measurements and the researcher was blinded for the clinical assessments of the doctors and nursing staff. All clinical assessments were stored in the patients' files and the study measurements were stored separately. After completing the inclusion of the predefined number of patients, the researchers were de-blinded for the clinical assessments.

Statistical analysis

Due to the exploratory nature of this study, a sample size calculation and power analysis was omitted. We aimed to include approximately 20 patients in this pilot study. Continuous variables were presented as mean ± standard deviation (SD). Categorical variables were presented as frequency with percentages. Data distribution was verified using the Shapiro-Wilk test. Depending on the data distribution, a paired t-test was used for parametric data or the Mann-Whitney U test was used, for non-parametric data. Receiver operating characteristic (ROC) curves were calculated on Deltascan measurements and CAM-ICU scores on each time point. Secondly ROC curves were computed on Deltascan measurements and the clinical diagnosis of a delirium (assessed by the attending ICU doctors). Values of p<0.05 were considered statistically significant. Statistical Package for Social Sciences (SPSS, Chicago, IL, USA, Version 20.0) was used for data preparation and for statistical analyses.

## Results

**Figure 4:**
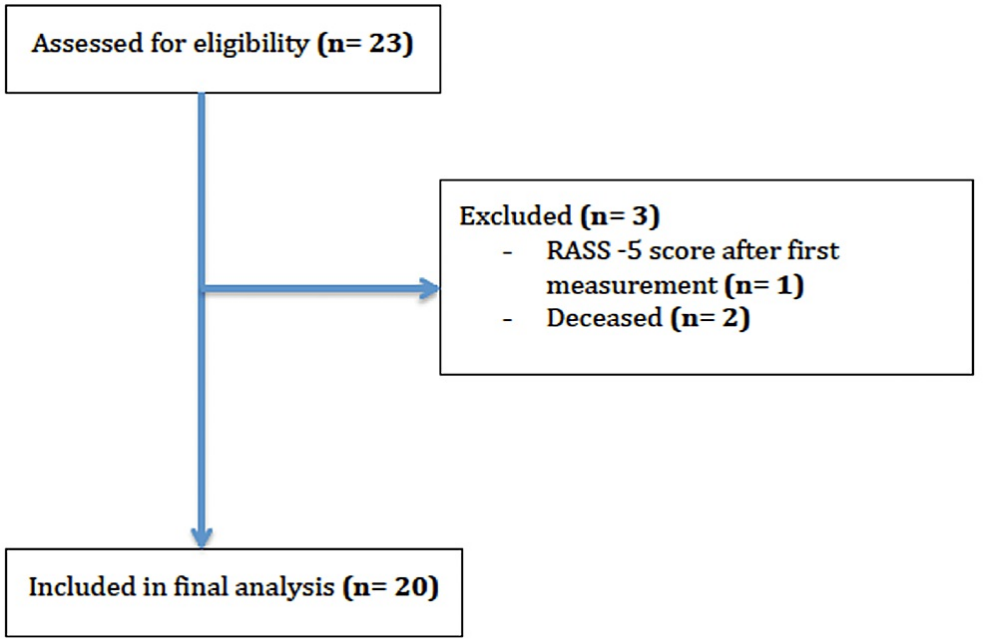
CONSORT diagram for the study

In total 23 patients were found eligible for this study, of which 20 were included in the final analyses. Of the three patients that were excluded, one of them had a RASS -5 level of consciousness after the first measurement, and the other two patients were deceased (Figure 4 for the CONSORT diagram).

Table 1 shows the baseline characteristics of the included patients. The mean age was 63.0 ± 8.8 years, and the majority (thirteen patients) were male (65%). Half of the included patients suffered from COVID-19 (n=10, 50%), and two patients were admitted with an oesophageal rupture due to an ulcer (n=2, 10%).

**Table 1:** Baseline characteristics of the included patients Abbreviations: BMI = Body Mass Index; COVID-19 = Coronavirus Disease-19, ICU = Intensive Care Unit

Patient characteristics	N= 20
Age (in years)	63.0 ± 8.8
Gender	
- Male	13 (65%)
- Female	7 (35%)
Anthropometric variables	
Length (in meter)	1.7 ± 0.09
Weight (in kilogram)	84.5 ± 15.3
BMI (in kg/m^2^)	29.0 ± 5.2
Diagnosis at ICU admittance	
Heart failure	1 (5.0%)
COVID-19	10 (50%)
Surgery for Gastric Cancer	1 (5.0%)
Anastomotic leakage after oesophagectomy	1 (5.0%)
Oesophageal rupture due to an ulcer	2 (10.0%)
Pneumonectomy	1 (5.0%)
Sepsis due to pneumonia	1 (5.0%)
Thoracic trauma	1 (5.0%)
Tracheal stenosis	1 (5.0%)
Nephrectomy due to urothelial carcinoma	1 (5.0%)

Deltascan and CAM-ICU measurements

Table 2 provides an overview of the Deltascan, CAM-ICU and RASS measurements per time point. In total 17 of the 20 patients (85%) received the diagnosis delirium by the medical team. There were no significant differences between the Deltascan and CAM-ICU measurements in diagnosing delirium per time point (p-values, respectively, 0.21; 0.90; 0.34; 0.11; 0.056 and 0.091).

**Table 2:** Deltascan, CAM-ICU and RASS measurements per time point Abbreviations: TP = Time point; CAM-ICU = Confusion Assessment Method-Intensive Care Unit; RASS = Richmond Agitation Sedation Scale; Nu = Nurse; Res= researcher

	Deltascan		CAM-ICU (Nu)		CAM-ICU (Res)		RASS	
	Score	N (%)	Score	N (%)	Score	N (%)	Score	N (%)
TP 1	0	1 (5)	1	17 (85)	1	16 (80)	-3	8 (40)
(N=20)	1	6 (30)	2	3 (15)	2	4 (20)	-2	4 (20)
Intubated: N=20	2	-					-1	4 (20)
	3	4 (20)					0	4 (20)
	4	4 (20)						
	5	4 (20)						
TP 2	0	1 (5)	1	17 (85)	1	17 (85)	-3	4 (20)
(N=20)	1	6 (30)	2	3 (15)	2	3 (15)	-2	6 (30)
Intubated: N=18	2	1 (5)					-1	3 (15)
	3	3 (15)					0	6 (30)
	4	3 (15)					+1	1 (5)
	5	6 (30)						
TP 3	0	4 (22.2)	1	14 (77.8)	1	12 (66.7)	-5	1 (5.6)
(N=18)	1	4 (22.2)	2	4 (22.2)	2	6 (33.3)	-3	2 (11.1)
Intubated: N=13	2	3 (16.7)					-2	4 (22.2)
	3	1 (5.6%)					-1	2 (11.1)
	4	2 (11.1)					0	8 (44.4)
	5	4 (22.2)					+2	1 (5.6)
TP 4	0	2 (14.3)	1	11 (78.6)	1	10 (71.4)	-3	2 (14.3)
(N=14)	1	3 (21.4)	2	3 (15.0)	2	3 (28.6)	-2	2 (14.3)
Intubated: N=12	2	2 (14.3)					-1	4 (28.6)
	3	-					0	4 (28.6)
	4	2 (14.3)					+1	2 (14.3)
	5	5 (35.7)						
TP 5	0	4 (36.4)	1	8 (72.7)	1	6 (54.5)	-3	2 (18.2)
(N=11)	1	1 (5.0)	2	3 (27.3)	2	5 (45.5)	-2	1 (9.1)
Intubated: N=7	2	-					-1	2 (18.2)
	3	1 (5.0)					0	4 (36.4)
	4	3 (15.0)					+1	2 (18.2)
	5	2 (10.0)						
TP 6	0	1 (16.7)	1	4 (66.7)	1	3 (50.0)	-3	1 (16.7)
(N=6)	1	2 (33.3)	2	2 (33.3)	2	3 (50.0)	-2	1 (16.7)
Intubated: N=5	2	-					-1	2 (33.3)
	3	-					0	1 (16.7)
	4	2 (33.3)					+1	1 (16.7)
	5	1 (16.7)						

Level of agreement between the Deltascan, CAM-ICU and clinical diagnosis of delirium

ROC curves were calculated to estimate the area under the curve (AUC) to determine the level of agreement between above-mentioned factors. For the level of agreement between the CAM-ICU and the Deltascan measurements the following AUCs were found: 0.676 ± 0.205 (Time point 1); 0.333 ± 0.224 (Time point 2); 0.402 ± 0.146 (Time point 3); 0.488 ± 0.202 (Time point 4); 0.06 ± 0.077 (Time point 5) and 0.06 ± 0.109 (Time point 6). None of them was statistically significant (p>0.05). When looking at the AUCs for level of agreement between the clinical diagnosis delirium and Deltascan we observed the following: 0.676 ± 0.152 (Time point 1); 0.686 ± 0.146 (Time point 2); 0.711 ± 0.132 (Time point 3); 0.688 ± 0.136 (Time point 4); 0.500 ± 0.158 (Time point 5) and 0.700 ± 0.211 (Time point 6). None of them was statistically significant (p>0.05).

## Discussion

In this exploratory pilot study, the feasibility of using a single channel EEG Deltascan measurement for detecting delirium in intubated ICU patients was investigated. The second aim of our study was to assess the level of agreement between the EEG measurements, the CAM-ICU score and the clinical diagnosis of delirium. The results showed that on several measurement points there is a reasonable yet non-significant agreement between both the CAM-ICU and Deltascan measurements and clinical delirium diagnoses and Deltascan measurements. This small single-center pilot study could be hypothesis-generating for further large-scale, possibly randomised clinical trials.

Especially in the ICU, delirium is a challenging diagnosis that is often missed [13]. Even with a previous extensively validated tool like the CAM-ICU, it still remains difficult to diagnose [13,15]. Diagnosing a delirium with the CAM-ICU was dependent on a variety of factors; of which the patient groups and nursing teams are the most important ones [14-16]. Irrespective of the patient population and training of nurses, the sensitivity varies between 40% and 80% in diagnosing delirium. One of the downsides of the CAM-ICU is that it can be difficult to use in patients that are deeply sedated [14-16]. This was shown in the study done by van Eck van der Sluijs et al. [13]. In this single centre study, a total of 103 ICU patients (a total of 502 days) were followed and the results showed that CAM-ICU was not usable for the patient with a high RASS score or a comatose state (N = 159 days [31.7%]). In our study, we found a repeatedly, yet non-significant AUC between the CAM-ICU and the Deltascan. There was a higher AUC repeatedly (also non-significant) between the Deltascan and the clinical diagnosis of delirium. It seems that there is more agreement between the Deltascan and the clinical diagnostics capacities of ICU physicians. However, this still needs to be confirmed with a larger study with more patients.

Since delirium is by definition, a fluctuating illness, with different presentations over time, there can be challenges in diagnosing [13,18,22]. Interestingly, the two patients who deceased during the study period had a high score on the Deltascan (four and five, respectively), indicating a high probability of delirium; they became septic approximately two days later. For these patients, the Deltascan picked up changes in polymorph delta waves, despite the fact that they were clinically not delirious (based on the ICU physician's observation and a negative CAM-ICU done by the ICU nurse). This indicates that a possible sepsis-induced very early changes in the brain can be possibly picked up by the Deltascan before they are clinically present. The pathophysiology of sepsis-associated delirium and related cognitive changes have a multifactorial pathophysiological basis [23]. Regarding the pathophysiology, several mechanisms have been described in the literature: direct microbial invasion, alterations in cerebral microcirculation, neurotransmission and metabolism [23-26]. Although the pathophysiology of sepsis-related delirium remains poorly understood it is thought that cerebral endothelial activation and breakdown of the blood-brain barrier (BBB) are the most important since they have been documented in both ‘in vivo’ and ‘in vitro’ animal models of the septic brain [25,26]. We postulate that due to early septic and immunological changes the BBB will break down, which causes a cerebral influx of inflammatory mediators. This will cause an inflammatory reaction and, can be picked up in the form of ‘polymorphic delta waves’ by the Deltascan. For follow-up studies in might be interesting to investigate whether the Deltascan can predict the occurrence of sepsis in ICU patients due to these early changes [27].

Limitations

Our study has limitations that need to be addressed. Firstly, this is a small single-center exploratory study, which might be underpowered to detect small statistically significant differences. Secondly, we have seen that since there is no ‘gold standard’ in assessing delirium in ICU patients that the different measurement methods have their own downsides. For example, the CAM-ICU does not always seem to detect a delirium with a sedation score of -3, this seems more likely to be the case with the Deltascan. In addition, the CAM-ICU is a questionnaire that can be interpreted differently. Finally, before usage of the Deltascan you must be adequately trained by an experienced user.

## Conclusions

In this exploratory pilot study, we showed that there is a moderate agreement between CAM-ICU and Delta scan measurements, but unfortunately not statistically significant. A similar tendency is seen in the agreement between the clinical diagnoses of delirium (by a clinician) with the Deltascan measurements. Despite this small study, we think that the Deltascan can be of additional value (for early delirium detection) in intubated ICU patients and therefore larger studies are needed to substantiate our findings.
